# Exploring Plasma-Level Gut Microbiota Mediators and Pro-Inflammatory Markers in Pregnant Women with Short Cervix and Gestational Diabetes Mellitus

**DOI:** 10.3390/ijms241713653

**Published:** 2023-09-04

**Authors:** Angela Silvano, Elena Niccolai, Simone Baldi, Viola Seravalli, Noemi Strambi, Giulia Nannini, Marco Pallecchi, Gianluca Bartolucci, Astrid Parenti, Amedeo Amedei, Mariarosaria Di Tommaso

**Affiliations:** 1 Department of Health Sciences, Division of Obstetrics and Gynecology, Careggi Hospital, University of Florence, 50139 Florence, Italy; angela.silvano@unifi.it (A.S.); viola.seravalli@unifi.it (V.S.); noemi.strambi@gmail.com (N.S.); 2 Department of Experimental and Clinical Medicine, University of Florence, 50134 Florence, Italy; elena.niccolai@unifi.it (E.N.); simone.baldi@unifi.it (S.B.); giulia.nannini@unifi.it (G.N.); amedeo.amedei@unifi.it (A.A.); 3 Department of Neurosciences, Psychology, Drug Research and Child Health, University of Florence, 50019 Sesto Fiorentino, Italy; marco.pallecchi@unifi.it (M.P.); gianluca.bartolucci@unifi.it (G.B.); 4 Department of Health Sciences, Clinical Pharmacology and Oncology Section, University of Florence, 50139 Florence, Italy

**Keywords:** preterm delivery, gestational diabetes mellitus, microbial translocation, lipopolysaccharide-binding protein, free fatty acids, MMP8, IL-8, Hsp70

## Abstract

The composition of the gut microbiota (GM) undergoes significant changes during pregnancy, influenced by metabolic status, energy homeostasis, fat storage, and hormonal and immunological modifications. Moreover, dysbiosis during pregnancy has been associated with preterm birth, which is influenced by factors such as cervical shortening, infection, inflammation, and oxidative stress. However, dysbiosis also affects the levels of lipopolysaccharide-binding protein (LBP), short-chain fatty acids (SCFAs), and free fatty acids (FFA) in other tissues and the bloodstream. In this study, we investigated the plasmatic levels of some pro-inflammatory cytokines, such as matrix metalloproteinases-8 (MMP-8), interleukin-8 (IL-8), heat shock protein 70 (Hsp70), and microbial markers in pregnant women with a short cervix (≤25 mm) compared to those with normal cervical length (>25 mm). We examined the differences in the concentration of these markers between the two groups, also assessing the impact of gestational diabetes mellitus. Understanding the relationship between GM dysbiosis, inflammatory mediators, and cervical changes during pregnancy may contribute to the identification of potential biomarkers and therapeutic targets for the prevention and management of adverse pregnancy outcomes, including preterm birth.

## 1. Introduction

The gut microbiota (GM) is a complex community of microorganisms that plays a crucial role in the development of the intestinal immune system [1] and is involved in various aspects of host metabolism, immunity, nutrition absorption and also the maintenance of a healthy pregnancy [2,3]. GM alterations, known as dysbiosis, can lead to increased permeability of the intestinal barrier, allowing microbial translocation to blood vessels and triggering systemic inflammatory processes [4]. Gut dysbiosis is implicated in the pathogenesis and development of various diseases, including obesity, type 2 diabetes, atherosclerosis, hypertension, chronic kidney diseases [5,6,7] and it is also involved in numerous immune-mediated disorders [8,9]. Dysbiosis also affects the levels of lipopolysaccharide-binding protein [10] (LBP), short-chain fatty acids (SCFA) [11,12] and free fatty acids (FFA) [13] in tissues outside the gut and in the bloodstream. LBP is a surrogate marker of microbial translocation, and it is higher in dysbiosis [14]. SCFAs, produced by anaerobic bacterial metabolism in the intestine, include butyric acid, propionic acid, acetic acid, valeric acid and caproic acid [15]. Their levels are influenced by diet, age, and other conditions, including obesity, hypertensive disorders and diabetes complicating pregnancy [15,16]. Other FFA are released from adipocytes as an energy source and circulate in blood as free or albumin-bound molecules. FFA exert immunomodulatory effects, mainly anti-inflammatory ones [17], such as the modulation of IL-18 levels via inflammasome NLRP3 activation and promoting T-cell differentiation via IL-10, IL-17 and IFN-γ levels. Moreover, FFA are associated with insulin resistance, endothelial dysfunction, obesity, type 2 diabetes mellitus and hypertension [18]. Assessing circulating FFA levels allows the investigation of systemic effects of GM on distant organs and on distal immune cells [19].

Gestational Diabetes Mellitus (GDM) contributes to changes in the composition and diversity of intestinal microorganisms, as well as altered proportions of FFA [20,21]. GDM is a common pregnancy disorder associated with adverse maternal and fetal outcomes [20]. The relationship between GDM and gut microbiota needs to be clarified. Indeed, some studies have investigated the intestinal microbiota during pregnancy and its changes according to the trimester of pregnancy, and the occurrence of GDM [22,23,24].

The composition of gut microbiota undergoes significant changes during gestation [25]. From the first to the third trimester of pregnancy, there is an increase in strains associated with inflammation (such as Bifidobacteria phylum, Proteobacteria phylum and lactic acid-producing bacteria), while the levels of *Faecalibacterium*, a butyrate-producing bacterium with anti- inflammatory activities, decrease [26].

These variations in GM depend on modification in metabolic status, energy homeostasis, fat storage, as well as hormonal and immunological changes that occur in healthy pregnancies [26]. In fact, the metabolic changes associated with pregnancy resemble those seen in the metabolic syndrome, including weight gain, elevated fasting blood glucose levels, insulin resistance, glucose intolerance, low-grade inflammation, and changes in metabolic hormone levels [27]. Additionally, immune changes during pregnancy ensure active immunological tolerance to the fetus, promoting its healthy development [28]. These immune changes are likely to have an impact on the microbiota [25,29]. Furthermore, it has been hypothesized that the GM dysbiosis during pregnancy may be associated with preterm birth [30]. The maternal microbiota, based on the ability to gain electrons, is believed to influence the maternal oxidative balance [31]. Moreover, oxidative stress is linked to adverse pregnancy outcomes, including preterm delivery [32,33].

The risk of spontaneous preterm birth is higher in women with a short cervical length [34,35] and genetic factors, uterine over-extension, infection and inflammation have all been associated with cervical shortening [36,37]. Elevated levels of pro-inflammatory mediators, such as matrix metalloproteinases-8 (MMP-8), interleukin-8 (IL-8), and heat shock protein 70 (Hsp70), in the vaginal fluid have been associated with cervical shortening [38,39]. Maternal plasmatic levels of IL-8 and Hsp70 have also been implicated in the pathogenesis of preterm birth [40,41,42]. However, the plasma levels of Hsp-70 and IL-8 have not been studied in pregnant women with short cervical length. Moreover, no study has evaluated maternal plasma levels of MMP-8 in women with cervical shortening, although increased MMP-8 concentration in the amniotic fluid has been observed in pregnant women with preterm labor [43].

Given this context, our study aims to compare the plasma concentration of pro-inflammatory and microbial markers in women with short and normal cervical length, while also evaluating the impact of gestational diabetes mellitus.

## 2. Results

The demographic and clinical characteristics of the patients are presented in Table 1. There were no significant differences observed in the maternal age, gestational age, and BMI at time of sampling between cases and controls. The median gestational age at delivery was also similar between the two groups. However, 11/64 women (17%) in the group with short cervical length delivered preterm, while all controls delivered at term.

### 2.1. Plasmatic Cytokines’ Determination

The results of the comparison of plasmatic cytokines between cases and controls are presented in Table 2. The plasmatic concentration of MMP-8 and Hsp70 was significantly lower in the case group compared to the controls (*p* < 0.0001). Conversely, IL-8 plasmatic concentration was higher in the case group compared to the controls (*p* < 0.001). There were no associations found between the levels of IL-8, MMP-8, or Hsp70 and gestational age at the time of enrolment, gestational age at delivery, BMI, or spontaneous preterm birth. However, we observed a positive correlation between cervical length and MMP-8 (Spearman’s Rho = 0.504, adj *p* < 0.001) and Hsp-70 (Spearman’s Rho = 0.367, adj *p* = 0.008) and a negative correlation with IL-8 (Spearman’s Rho = −0.278, adj *p* = 0.002).

In our cohort, 15 pregnant women with a short cervix and 10 pregnant women with normal cervical length were diagnosed with GDM but the plasmatic concentration of MMP-8, IL-8 and Hsp70 did not significantly differ between women with and without GDM in either group (Table 3).

### 2.2. Plasmatic LBP Protein Dosage

The median concentration of LBP was 19.60 µg/mL (15.90–24.00) in the case group and 22.63 µg/mL (15.10–26.60) in the control group, but the difference was not statistically significant (Table 2). Moreover, no difference was observed between patients with or without GDM (Table 3).

### 2.3. Evaluation of the Plasmatic Free Fatty Acids

The median and percentile values of each FFA in the different study groups are reported in Table 2 and Table 3. The levels of FFAs in the different groups exhibited large intra-group variability, with no significant differences observed. Furthermore, no association was found between levels of FFA and cervical length, gestational age at the time of enrolment, gestational age at delivery, BMI, or spontaneous preterm birth.

## 3. Materials and Methods

### 3.1. Study Design

This prospective study was conducted between 2016 and 2020 at the Department of Obstetrics and Gynecology of Careggi University Hospital in Florence, Italy. The study enrolled a total of 89 pregnant women, of which 64 had a short cervix (≤25 mm), and 25 had a normal cervical length, and served as controls. Both groups included women with gestational diabetes mellitus (GDM): fifteen pregnant women with cervical length ≤ 25 mm and GDM (case group with GDM) and ten pregnant women with normal cervical length and GDM (control group with GDM). We included diet-controlled GDM cases as well as those requiring insulin treatment.

The cases with short cervix were referred to the Hospital’s Preterm Birth Clinic by their obstetricians who detected cervical shortening on a transvaginal ultrasound performed during a routine prenatal visit. Controls were selected among women attending the Hospital’s Maternity Outpatient Clinic for routine prenatal visits. Gestational age was calculated from the last menstrual period and confirmed by fetal crown-rump length measurement from the first trimester ultrasound. Exclusion criteria for the study were as follows: multiple pregnancy, a previous surgery to the cervix, evidence of premature rupture of membranes or symptomatic uterine contractions at the time of recruitment, vaginal infection at the time of recruitment, the presence of a cervical cerclage or pessary. There were no cases of chronic comorbidities, anemia and preeclampsia in our study cohort. There were two cases of fetal growth restriction, one for each group, that were diagnosed in the third trimester.

### 3.2. Cervical Length Measurment

The measurement of the cervical length was performed by transvaginal ultrasound, after the patients had emptied their bladder, according to the standard technique. On the sagittal plane, the full length of the endocervical canal was visualized and the distance between the internal to the external cervical was measured. At least three measurements were obtained and the shortest measurement was recorded.

### 3.3. Diagnosis of GDM

GDM was diagnosed based on fasting plasma glucose ≥ 5.1 mmol/L, and/or 1-h plasma glucose ≥ 10.0 mmol/L, and/or 2-h plasma glucose ≥ 8.6 mmol/L in the 75 g oral glucose tolerance test [44].

### 3.4. Collection of Plasma Samples

Six mL blood samples were collected by venipuncture into tubes containing EDTA and centrifuged at 2000 RPM for 10 min. One ml of plasma was stored at −80 °C.

### 3.5. LBP Protein Dosage

Lipopolysaccharide binding protein (LBP) level was quantified in plasma samples by Human-LBP ELISA kit, following manufacturer’s instructions (Hycult Biotech, Uden, The Netherlands).

### 3.6. Free Fatty Acids Evaluation

The plasma levels of short, medium and long chain fatty acids were measured using Gas Chromatography coupled with Mass Spectrometry. The preparation of standard curves was carried out using an Agilent GC-MS 114 system, which consisted of a 5971 single quadrupole mass spectrometer, 5890 gas chromato-115 graph and 7673 autosampler. The details of the methodology can be found in a previous publication [45].

### 3.7. Cytokines’ Plasma Determination

For the determination of MMP8, IL-8 and Hsp70 levels in plasma, a Milliplex custom kit Human Sepsis Panel 2 Magnetic Bead Panel for Luminex MAGPIX detection system (Affymetrix, eBioscience, Santa Clara, CA, USA) was used. The manufacturer’s instructions for plasma samples were followed during the analysis of cytokines.

### 3.8. Ethics Approval

Ethics approval for this study was granted by the local ethics committee of Careggi University Hospital, Florence (Ref. no. BIO14.0009-09/07/2014), and all women provided written informed consent.

### 3.9. Statistical Analysis

The levels of the plasmatic markers were compared between the group of cases and controls using the independent samples Mann–Whitney test for continuous variables and with the χ^2^ test for unpaired data for categorical variables. A subgroup analysis was also conducted to compare the concentrations of the markers between women with and without GDM within each group. Continuous variables were reported by medians and interquartile range (IQR), while categorical ones by absolute and relative frequencies. Spearman correlation coefficients were computed to assess association between variables. The statistical analysis was performed using the IBM SPSS Statistics, version 28. A *p* < 0.05 was considered statistically significant. *p*-values were corrected for multiple comparisons using the Bonferroni correction.

## 4. Discussion

The present study indicates that pregnant women with normal cervical length (>25 mm) have higher plasmatic concentrations of MMP-8 and Hsp70 compared to women with a short cervix (≤25 mm). Notably, the levels of these markers showed a significant correlation with cervical length and remained consistent regardless of GDM status. To the best of our knowledge, this is the first study to demonstrate a correlation between plasmatic MMP-8 and Hsp-70 levels and the length of the uterine cervix during pregnancy.

MMP-8, also known as collagenase-2 or neutrophil collagenase, is expressed in various cell types, including peripheral neutrophils, macrophages, plasma cells and T cells [46,47]. MMP-8 and other MMPs play crucial roles in cervical remodelling, and their concentration and activity have been reported to increase in the lower uterine segment during labor [48]. We hypothesize that the lower plasma MMP-8 levels observed in pregnant women with a short cervix may be associated with higher levels of this enzyme in the placenta or in the lower uterine segment, where inflammation and remodelling may occur [49]. MMP-8 has been shown to compromise epithelial barrier integrity [47] and its levels in vaginal fluid have been associated with cervical shortening [39]. Elevated MMP-8 concentration in amniotic fluid has been also linked to preterm birth and preterm premature rupture of membranes [50,51]. However, no association has been found between cervical fluid MMP-8 concentration and preterm birth in early and mid-pregnancy [52].

With regard to Hsp70, previous studies have reported elevated levels in the serum of women with preeclampsia compared to those with normal pregnancy [53,54], as well as in vaginal fluid of women with short cervix, suggesting its involvement in the pro-inflammatory cervico-vaginal milieu leading to cervical shortening [39]. The lower plasmatic concentration of Hsp70 observed in women with short cervix in this study may be attributed to the recall of the pro-inflammatory cells and molecules to the shortening cervix, where they play a role in the cervical tissue remodelling [39,49,55].

Finally, the IL-8, also known as CXCL-8, is a pro-inflammatory chemokine with a primary function in attracting and activating neutrophils, and is involved in cellular processes in numerous pregnancy-related events [56,57]. Interestingly, IL-8 can influence cervical MMP activity, promoting the release of MMP-8 and MMP-9 from neutrophils [58].

Elevated maternal IL-8 serum has been observed in patients with chorioamnionitis compared to those without chorioamnionitis [59], as well as in vaginal fluid of pregnant women with short cervix [39]. In our study, we found a higher concentration of plasmatic IL-8 in pregnant women with a short cervix. It has been reported that plasmatic IL-8 tends to increase between the second and the third trimester of physiological pregnancies [60], indicating a shift towards a pro-inflammatory systemic profile as the parturition approaches [57,61].

To explore the potential association between the risk of preterm delivery in women with a short cervix and GM dysbiosis, we evaluated the plasma levels of LBP and various short, medium and long chain fatty acids. To our knowledge, this study represents the first attempt to assess these parameters in pregnant women with a shortened uterine cervix.

The integrity of the gut mucosal barrier is crucial for maintaining immune homeostasis by preventing the entry of harmful pathogens into the system. However, when the barrier integrity is compromised it can result in excessive microbial translocation, leading to immune activation and the development of different diseases, such as type II diabetes and infections [62,63].

LBP is a protein that specifically binds to lipopolysaccharide (LPS), enhancing its interaction with the immune system [64], and serves as a reliable marker for microbial translocation. Elevated LBP levels have been observed in several inflammatory conditions [14,65]. Pregnancy is associated with increased gut permeability, which can lead to metabolic alterations that predispose to insulin resistance [66,67]. The combination of increased permeability, bacterial translocation and maternal gut dysbiosis, can increase the risk of various pregnancy complications, including gestational diabetes, preeclampsia, and preterm birth [68,69,70]. In mice implanted with intestinal microflora from women in the third trimester of gestation, increases in body weight and insulin resistance were observed, reflecting the diabetogenic changes observed in pregnant women. The results reported by Koren et al. may indicate that changes in the intestinal microflora during pregnancy contribute to the occurrence of metabolic changes characteristic of pregnancy, with an increase in inflammatory markers and energy content [26].

Therefore, measuring LBP levels in pregnant women may serve as an indicator of microbial translocation and identify those at an increased risk of adverse pregnancy outcomes. Limited evidence exists regarding the role of LPS-related molecules in pregnancy, with few studies assessing LBP specifically. However, some studies have reported increased LBP levels in the amniotic fluid of women with preterm labor or intrauterine infection [71], as well as elevated levels of LPS in cord blood of preterm births compared to term pregnancies, particularly in cases with more-severe chorioamnionitis in the placenta [72]. Additionally, elevated LBP levels have been observed in pregnant women infected with HIV in the first trimester compared to non-infected individuals, with an association between increased levels and premature delivery [73].

In our study, the median LBP levels in women with a short cervix were similar to those in the control group, suggesting that cervical length may not be associated with LBP levels. No correlation was found between LBP levels and BMI and, unexpectedly, no association was observed between GDM and LBP levels. It is possible that factors such as differences in the severity or duration of GDM or variations in the GM composition among women, may have influenced the results. Further studies involving larger cohorts of patients and controlling for potential confounding factors are necessary to examine the relationship between GDM and LBP levels more effectively.

In our study, we aimed to assess the presence of functional dysbiosis in pregnant women with a short cervix by evaluating circulating FFA levels.

Free fatty acids have been associated with inflammation and oxidative stress, and their assessment in cases of cervical shortening could provide insights into the underlying mechanisms of preterm birth and the identification of new risk biomarkers for preterm delivery, with the aim of developing preventive strategies. Previous research has shown that elevated fasting plasma FFA levels at 30 weeks’ gestation are associated with an increased risk of preterm delivery [74]. However, our data showed no significant differences in FFA levels between pregnant women with a short cervix and controls. Additionally, no significant differences in FFA levels were observed between pregnant women with and without gestational diabetes, contrary to findings from previous studies [21]. It is important to note that the gestational age and BMI at sampling were similar between cases and controls in our study, which helps to control for potential confounding effects of these factors on FFA levels. It is known FFA circulating levels can vary throughout gestation [75].

Overall, our findings provide new insights into the association between cervical shortening and the systemic levels of pro-inflammatory and microbial markers. However, the results indicate that LBP and FFA levels may not be associated with cervical shortening or gestational diabetes. The limitations of our study include the lack of different ethnic groups in our study cohort, which did not allow to study the effect of ethnicity on inflammatory and microbial markers, and the relatively small sample size.

Further studies with larger sample sizes and different study populations are necessary to confirm these findings and explore other potential factors that may affect FFA levels in pregnant women.

## 5. Conclusions

In conclusion, this study sheds light on the intricate connections between cervical length, systemic markers of inflammation, and gut microbiota-related parameters in pregnant individuals. The novel findings linking plasma levels of MMP-8, Hsp70, and IL-8 to cervical length provide insights into the complex processes of cervical remodeling and potential preterm birth risk. The association between these markers and the length of the uterine cervix regardless of GDM status underscores their potential significance as universal indicators of cervical health. Additionally, while this study did not find direct correlations between LBP and FFA levels with cervical shortening or GDM, these parameters still remain valuable to explore in larger and more diverse cohorts to establish a comprehensive understanding of their roles in pregnancy complications. Altogether, these findings contribute to the broader understanding of maternal physiology during pregnancy and the intricate interplay between various factors that influence maternal and fetal well-being. Further investigations are warranted to uncover the full spectrum of connections and implications arising from these intriguing observations.

## Figures and Tables

**Table 1:** Patient demographic and clinical information.

Clinical Characteristics	All Pregnant Women	Pregnant Women with Short Cervix ≤ 25 mm	Pregnant Women with Cervix > 25 mm	*p* Value
Number of enrolled pregnant women	89	64	25	
Ethnicity				1.000
Caucasian	88	63	25	
Asian	1	1	0	
Age at sampling (years)				
mean ± SD	33.2 ± 6.8	33.1 ± 7.0	33.4 ± 6.5	0.985
BMI kg/m^2^				
mean ± SD	23.70 ± 5.3	23.45 ± 5.3	24.24 ± 5.4	0.467
Gestational diabetes mellitus	25 (28%)	15 (23.4%)	10 (40%)	0.118
Gestational age at sampling (weeks)				
mean ± SD	28.4 ± 2.4	28.3 ± 2.2	28.5 ± 3.0	0.919
Length of the cervix (mm) at sampling				
mean ± SD	19.6 ± 9.0	15.1 ± 5.8	31.0 ± 4.7	<0.001
Gestational age at birth (weeks)				
mean ± SD	38.1 ± 2.4	38.0 ± 2.7	38.6 ± 1.5	0.562
Spontaneous preterm birth	11 (12.4%)	11 (17.0%)	0 (0%)	0.030

**Table 2:** Plasmatic concentrations of cytokines, LBP, and free fatty acids in the case and control group. Pregnant women with short cervix are indicated as cases group. Pregnant women with normal cervical length as control group. Values are reported as median and interquartile range (IQR). *p*-values were assessed with the independent samples Mann–Whitney test (Bonferroni correction). * adj *p*-value < 0.002.

Analytes	Case Group (n = 64)	Control Group (n = 25)	*p* Value
MMP-8 (pg/mL)	848.60 (524.55–1283.61)	3351.55 (1259.20–6426.03)	<0.001 *
Hsp70 (ng/mL)	66.42 (51.45–83.90)	138.00 (81.00–167.60)	<0.001 *
IL-8 (pg/mL)	4.00 (3.10–6.26)	2.60 (1.80–3.70)	<0.001 *
LBP (µg/mL)	19.60 (15.90–24.00)	22.63 (15.10–26.60)	0.471
Acetic acid (µmol/L)	115.08 (105.71–129.79)	119.50 (111.58–129.75)	0.273
Propionic acid (µmol/L)	2.03 (1.89–2.57)	1.89 (1.49–2.84)	0.540
Butyric acid (µmol/L)	0.74 (0.57–1.11)	0.68 (0.57–0.86)	0.150
Iso-Butyric acid (µmol/L)	7.05 (6.93–7.16)	7.16 (7.05–7.27)	0.007
Iso-Valeric acid (µmol/L)	0.20 (0.20–0.29)	0.29 (0.20–0.29)	0.136
2-MethylButyric acid (µmol/L)	0.20 (0.20–0.20)	0.20 (0.20–0.29)	0.771
Valeric acid (µmol/L)	0.20 (0.10–0.20)	0.20 (0.15–0.20)	0.045
IsoHexanoic acid (µmol/L)	0.69 (0.69–0.78)	0.69 (0.69–0.74)	0.132
Hexanoic acid (µmol/L)	0.34 (0.34–0.50)	0.34 (0.30–0.52)	0.732
2-Ethyl-Hexanoic acid (µmol/L)	2.57 (2.50–2.57)	2.57 (2.50–2.57)	0.019
Octanoic acid (µmol/L)	4.10 (3.89–4.44)	3.89 (3.79–4.62)	0.574
Decanoic acid (µmol/L)	4.27 (3.79–5.32)	4.71 (4.13–5.61)	0.152
Dodecanoic acid (µmol/L)	2.93 (2.35–3.55)	2.85 (2.40–3.73)	0.722
Tetradecanoic acid (µmol/L)	16.45 (13.60–22.17)	15.96 (12.90–21.32)	0.391
Hexadecanoic acid (µmol/L)	497.75 (404.24–707.49)	526.91 (385.76–642.01)	0.729
Octadecanoic acid (µmol/L)	172.78 (141.42–247.59)	179.33 (152.35–229.81)	0.869

**Table 3:** Plasmatic levels of cytokines, LBP, and free fatty acids in women with and without gestational diabetes mellitus (GDM) in the case and control group. Median and interquartile range (IQR) were reported. *p* values were assessed with the independent samples Mann–Whitney test (Bonferroni correction).

Analytes	Case Group No GDM (n = 49)	Case Group GDM (n = 15)	*p* Values	Control Group No GDM (n = 15)	Control Group GDM (n = 10)	*p* Values
MMP-8 (pg/mL)	838.96 (530.00–1249.70)	865.10 (348.61–1341.20)	0.930	1818.00 (1038.23–4987.00)	3463.65 (1960.51–7227.65)	0.311
Hsp70 (ng/mL)	65.93 (46.70–83.40)	71.00 (52.41–85.85)	0.794	97.90 (68.60–159.20)	144.25 (136.82–179.00)	0.437
IL-8 (pg/mL)	4.30 (2.80–5.30)	5.00 (4.30–9.80)	0.058	2.40 (1.90–3.30)	3.30 (1.61–7.30)	0.080
LBP (µg/mL)	19.21 (3.59–24.37)	19.75 (13.48–23.74)	0.831	23.84 (15.91–29.27)	18.54 (12.18–23.54)	0.311
Acetic acid (µmol/L)	114.17 (5.17–130.50)	121.00 (110.17–127.50)	0.338	119.50 (112.17–129.33)	119.92 (109.63–141.92)	0.935
Propionic acid (µmol/L)	2.03 (4.76–2.43)	2.43 (2.03–2.70)	0.030	2.16 (1.62–3.11)	1.83 (1.46–2.83)	0.144
Butyric acid (µmol/L)	0.68 (0.57–1.02)	1.02 (0.57–1.14)	0.306	0.68 (0.57–0.80)	0.63 (0.57–0.63)	0.849
Iso-Butyric acid (µmol/L)	7.05 (1.93–7.16)	7.05 (6.93–7.27)	0.108	7.16 (7.05–7.27)	7.22 (7.05–7.22)	0.177
Iso-Valeric acid (µmol/L)	0.20 (2.20–0.29)	0.20 (0.20–0.39)	0.734	0.20 (0.20–0.29)	0.29 (0.18–0.29)	0.723
2-MethylButyric acid (µmol/L)	0.20 (2.20–0.20)	0.20 (0.20–0.29)	0.794	0.20 (0.20–0.29)	0.20 (0.18–0.20)	0.723
Valeric acid (µmol/L)	0.20 (2.10–0.20)	0.20 (0.10–0.20)	0.555	0.20 (0.20–0.20)	0.20 (0.10–0.20)	0.531
IsoHexanoic acid (µmol/L)	0.69 (7.69–0.78)	0.69 (0.69–0.78)	0.786	0.69 (0.69–0.69)	0.69 (0.67–0.69)	1.000
Hexanoic acid (µmol/L)	0.34 (4.34–0.43)	0.34 (0.26–0.52)	0.623	0.34 (0.26–0.52)	0.34 (0.32–0.34)	0.892
2-Ethyl-Hexanoic acid (µmol/L)	2.57 (5.50–2.57)	2.57 (2.50–2.57)	0.688	2.57 (2.50–2.57)	2.54 (2.50–2.54)	0.428
Octanoic acid (µmol/L)	4.03 (4.89–4.48)	4.10 (3.82–4.38)	0.886	3.89 (3.75–4.93)	3.93 (3.77–4.93)	0.807
Decanoic acid (µmol/L)	4.30 (4.87–5.44)	4.24 (3.66–5.35)	0.634	4.59 (4.13–5.52)	4.80 (4.10–6.80)	0.723
Dodecanoic acid (µmol/L)	3.00 (5.45–3.58)	2.50 (2.25–3.35)	0.231	3.05 (2.45–3.70)	2.80 (2.21–4.80)	0.765
Tetradecanoic acid (µmol/L)	16.93 (8.95–22.81)	15.61 (13.55–21.97)	0.516	16.62 (13.95–21.18)	13.40 (11.98–21.40)	0.285
Hexadecanoic acid (µmol/L)	502.19 (9.44–723.96)	491.02 (416.60–600.16)	0.981	542.62 (383.55–621.05)	473.50 (393.04–703.50)	0.643
Octadecanoic acid (µmol/L)	177.54 (4.78–250.42)	170.49 (130.39–182.78)	0.547	187.22 (158.84–216.69)	174.00 (145.04–248.00)	0.765

## Data Availability

Not applicable.

## References

[1] D’Amelio P., Sassi F. (2018). Gut Microbiota, Immune System, and Bone. Calcif. Tissue Int..

[2] Viennois E., Chassaing B. (2018). First victim, later aggressor: How the intestinal microbiota drives the pro-inflammatory effects of dietary emulsifiers?. Gut Microbes.

[3] Wang J., Gu X., Yang J., Wei Y., Zhao Y. (2019). Gut Microbiota Dysbiosis and Increased Plasma LPS and TMAO Levels in Patients With Preeclampsia. Front. Cell Infect. Microbiol..

[4] Natividad J.M.M., Verdu E.F. (2013). Modulation of intestinal barrier by intestinal microbiota: Pathological and therapeutic implications. Pharmacol. Res..

[5] Andersen K., Kesper M.S., Marschner J.A., Konrad L., Ryu M., Kumar Vr S., Kulkarni O.P., Mulay S.R., Romoli S., Demleitner J. (2017). Intestinal dysbiosis, barrier dysfunction, and bacterial translocation account for CKD-related systemic inflammation. J. Am. Soc. Nephrol..

[6] Jonsson A.L., Bäckhed F. (2017). Role of gut microbiota in atherosclerosis. Nat. Rev. Cardiol..

[7] Li J., Zhao F., Wang Y., Chen J., Tao J., Tian G., Wu S., Liu W., Cui Q., Geng B. (2017). Gut microbiota dysbiosis contributes to the development of hypertension. Microbiome.

[8] Kamada N., Núñez G. (2013). Role of the Gut Microbiota in the Development and Function of Lymphoid Cells. J. Immunol..

[9] Levy M., Kolodziejczyk A.A., Thaiss C.A., Elinav E. (2017). Dysbiosis and the immune system. Nat. Rev. Immunol..

[10] Lim P.S., Chang Y.K., Wu T.K. (2019). Serum Lipopolysaccharide-Binding Protein is Associated with Chronic Inflammation and Metabolic Syndrome in Hemodialysis Patients. Blood Purif..

[11] Morrison D.J., Preston T. (2016). Formation of short chain fatty acids by the gut microbiota and their impact on human metabolism. Gut Microbes.

[12] Hu M., Eviston D., Hsu P., Mariño E., Chidgey A., Santner-Nanan B., Wong K., Richards J.L., Yap Y.-A., Collier F. (2019). Decreased maternal serum acetate and impaired fetal thymic and regulatory T cell development in preeclampsia. Nat. Commun..

[13] Rodríguez-Carrio J., Salazar N., Margolles A., González S., Gueimonde M., Reyes-Gavilán C.G.d.L., Suárez A. (2017). Free fatty acids profiles are related to gut microbiota signatures and short-chain fatty acids. Front. Immunol..

[14] Stehle J.J.R., Leng X., Kitzman D.W., Nicklas B.J., Kritchevsky S.B., High K.P. (2012). Lipopolysaccharide-binding protein, a surrogate marker of microbial translocation, is associated with physical function in healthy older adults. J. Gerontol.-Ser. A Biol. Sci. Med. Sci..

[15] Ziętek M., Celewicz Z., Szczuko M. (2021). Short-Chain Fatty Acids, Maternal Microbiota and Metabolism. Nutrients.

[16] Lin H.V., Frassetto A., Kowalik E.J., Nawrocki A.R., Lu M.M., Kosinski J.R., Hubert J.A., Szeto D., Yao X., Forrest G. (2012). Butyrate and propionate protect against diet-induced obesity and regulate gut hormones via free fatty acid receptor 3-independent mechanisms. PLoS ONE.

[17] Ratajczak W., Rył A., Mizerski A., Walczakiewicz K., Sipak O., Laszczyńska M. (2019). Immunomodulatory potential of gut microbiome-derived shortchain fatty acids (SCFAs). Acta Biochim. Pol..

[18] Ghosh A., Gao L., Thakur A., Siu P.M., Lai C.W.K. (2017). Role of free fatty acids in endothelial dysfunction. J. Biomed. Sci..

[19] Schroeder B.O., Bäckhed F. (2016). Signals from the gut microbiota to distant organs in physiology and disease. Nat. Med..

[20] Dualib P.M., Ogassavara J., Mattar R., da Silva E.M.K., Dib S.A., Pititto B.D.A. (2021). Gut microbiota and gestational Diabetes Mellitus: A systematic review. Diabetes Res. Clin. Pract..

[21] Villafan-Bernal J.R., Acevedo-Alba M., Reyes-Pavon R., Diaz-Parra G.A., Lip-Sosa D.L., Vazquez-Delfin H.I., Hernandez-Muñoz M., Bravo-Aguirre D.E., Figueras F., Martinez-Portilla R.J. (2019). Plasma Levels of Free Fatty Acids in Women with Gestational Diabetes and Its Intrinsic and Extrinsic Determinants: Systematic Review and Meta-Analysis. J. Diabetes Res..

[22] Kuang Y.-S., Lu J.-H., Li S.-H., Li J., Yuan M.-Y., He J.R., Chen N.-N., Xiao W.-Q., Shen S.-Y., Qiu L. (2017). Connections between the human gut microbiome and gestational diabetes mellitus. Gigascience.

[23] Chen T., Zhang Y., Zhang Y., Shan C., Zhang Y., Fang K., Xia Y., Shi Z. (2021). Relationships between gut microbiota, plasma glucose and gestational diabetes mellitus. J. Diabetes Investig..

[24] Wang J., Zheng J., Shi W., Du N., Xu X., Zhang Y., Ji P., Zhang F., Jia Z., Wang Y. (2018). Dysbiosis of maternal and neonatal microbiota associated with gestational diabetes mellitus. Gut.

[25] Nuriel-Ohayon M., Neuman H., Koren O. (2016). Microbial Changes during Pregnancy, Birth, and Infancy. Front. Microbiol..

[26] Koren O., Goodrich J.K., Cullender T.C., Spor A., Laitinen K., Bäckhed H.K., Gonzalez A., Werner J.J., Angenent L.T., Knight R. (2012). Host remodeling of the gut microbiome and metabolic changes during pregnancy. Cell.

[27] Priyadarshini M., Thomas A., Reisetter A.C., Scholtens D.M., Wolever T.M., Josefson J., Layden B.T. (2014). Maternal short-chain fatty acids are associated with metabolic parameters in mothers and newborns. Transl. Res..

[28] Silvano A., Seravalli V., Strambi N., Cecchi M., Tartarotti E., Parenti A., Di Tommaso M. (2021). Tryptophan metabolism and immune regulation in the human placenta. J. Reprod. Immunol..

[29] Miko E., Csaszar A., Bodis J., Kovacs K. (2022). The Maternal–Fetal Gut Microbiota Axis: Physiological Changes, Dietary Influence, and Modulation Possibilities. Life.

[30] Baldassarre M.E., Di Mauro A., Capozza M., Rizzo V., Schettini F., Panza R., Laforgia N. (2019). Dysbiosis and prematurity: Is there a role for probiotics?. Nutrients.

[31] Cömert T.K., Akpinar F., Erkaya S., Durmaz B., Durmaz R. (2022). The effect of gestational weight gain on serum total oxidative stress, total antioxidant capacity and gut microbiota. Biosci. Microbiota. Food Health.

[32] Menon R. (2014). Oxidative stress damage as a detrimental factor in preterm birth pathology. Front. Immunol..

[33] Moore T.A., Ahmad I.M., Zimmerman M.C. (2018). Oxidative Stress and Preterm Birth: An Integrative Review. Biol. Res. Nurs..

[34] Iams J.D., Goldenberg R.L., Meis P.J., Mercer B.M., Moawad A., Das A., Thom E., McNellis D., Copper R.L., Johnson F. (1996). The length of the cervix and the risk of spontaneous premature delivery. National Institute of Child Health and Human Development Maternal Fetal Medicine Unit Network. N. Engl. J. Med..

[35] Di Tommaso M., Berghella V. (2013). Cervical length for the prediction and prevention of preterm birth. Expert. Rev. Obstet. Gynecol..

[36] Romero R., Dey S.K., Fisher S.J. (2014). Preterm labor: One syndrome, many causes. Science.

[37] Lannon S.M.R., Vanderhoeven J.P., Eschenbach D.A., Gravett M.G., Waldorf K.M.A. (2014). Synergy and interactions among biological pathways leading to preterm premature rupture of membranes. Reprod. Sci..

[38] Jung E.Y., Park J.W., Ryu A., Lee S.Y., Cho S.-H., Park K.H. (2016). Prediction of impending preterm delivery based on sonographic cervical length and different cytokine levels in cervicovaginal fluid in preterm labor. J. Obstet. Gynaecol. Res..

[39] Sisti G., Paccosi S., Parenti A., Seravalli V., Linari C., Di Tommaso M., Witkin S. (2020). Pro-infl ammatory mediators in vaginal fluid and short cervical length in pregnancy. Bratisl. Med. J..

[40] Molvarec A., Tamási L., Losonczy G., Madách K., Prohászka Z., Rigó J. (2010). Circulating heat shock protein 70 (HSPA1A) in normal and pathological pregnancies. Cell Stress. Chaperones.

[41] Kim S.Y., Park K.H., Kim H.J., Kim Y.M., Ahn K., Lee K. (2022). Inflammation-related immune proteins in maternal plasma as potential predictive biomarkers for rescue cerclage outcome in women with cervical insufficiency. Am. J. Reprod. Immunol..

[42] Chen Y.S. (2022). The Association of Prenatal C-Reactive Protein and Interleukin-8 Levels with Maternal Characteristics and Preterm Birth. Am. J. Perinatol..

[43] Park C.W., Yoon B.H., Park J.S., Jun J.K. (2013). A Fetal and an Intra-Amniotic Inflammatory Response Is More Severe in Preterm Labor than in Preterm PROM in the Context of Funisitis: Unexpected Observation in Human Gestations. PLoS ONE.

[44] World Health Organization (2013). Diagnostic Criteria and Classification of Hyperglycaemia First Detected in Pregnancy.

[45] Baldi S., Pagliai G., Dinu M., Di Gloria L., Nannini G., Curini L., Pallecchi M., Russo E., Niccolai E., Danza G. (2022). Effect of ancient Khorasan wheat on gut microbiota, inflammation, and short-chain fatty acid production in patients with fibromyalgia. World J. Gastroenterol..

[46] Wahlgren J., Sorsa T., Sutinen M., Tervahartiala T., Teronen O., Hietanen J., Salo T., Maisi P., Pirilä E., Tjäderhane L. (2001). Expression and induction of collagenases (MMP-8 and -13) in plasma cells associated with bone-destructive lesions. J. Pathol..

[47] Van Lint P., Libert C. (2006). Matrix metalloproteinase-8: Cleavage can be decisive. Cytokine Growth Factor. Rev..

[48] Ledingham M.A., Denison F.C., Riley S.C., Norman J.E. (1999). Matrix metalloproteinases-2 and -9 and their inhibitors are produced by the human uterine cervix but their secretion is not regulated by nitric oxide donors. Hum. Reprod..

[49] Socha M.W., Flis W., Pietrus M., Wartęga M., Stankiewicz M. (2022). Signaling Pathways Regulating Human Cervical Ripening in Preterm and Term Delivery. Cells.

[50] Biggio J.R., Ramsey P.S., Cliver S.P., Lyon M.D., Goldenberg R.L., Wenstrom K.D. (2005). Midtrimester amniotic fluid matrix metalloproteinase-8 (MMP-8) levels above the 90th percentile are a marker for subsequent preterm premature rupture of membranes. Am. J. Obstet. Gynecol..

[51] Nien J.K., Yoon B.H., Espinoza J., Kusanovic J.P., Erez O., Soto E., Richani K., Gomez R., Hassan S., Mazor M. (2006). A rapid MMP-8 bedside test for the detection of intra-amniotic inflammation identifies patients at risk for imminent preterm delivery. Am. J. Obstet. Gynecol..

[52] Rahkonen L., Rutanen E.M., Nuutila M., Sainio S., Sorsa T., Paavonen J. (2010). Matrix metalloproteinase-8 in cervical fluid in early and mid pregnancy: Relation to spontaneous preterm delivery Leena. Prenat. Diagn..

[53] Molvarec A., Derzsy Z., Kocsis J., Bőze T., Nagy B., Balogh K., Makó V., Cervenak L., Mézes M., Karádi I. (2009). Circulating anti-heat-shock-protein antibodies in normal pregnancy and preeclampsia. Cell Stress. Chaperones.

[54] Romão-Veiga M., Matias M.L., Ribeiro V.R., Nunes P.R., Borges V.T.M., Peraçoli J.C., Peraçoli M.T.S. (2018). Induction of systemic inflammation by hyaluronan and hsp70 in women with pre-eclampsia. Cytokine.

[55] Volpato L.K., Nadkarni S., Horevicz V.V., Donatello N., Parma G.O.C., Martins D.F., Piovezan A.P. (2022). Contribution of plasma, placental, inflammatory and pro-resolving mediators in labor induction. Placenta.

[56] Li A., Dubey S., Varney M.L., Dave B.J., Singh R.K. (2003). IL-8 Directly Enhanced Endothelial Cell Survival, Proliferation, and Matrix Metalloproteinases Production and Regulated Angiogenesis. J. Immunol..

[57] Vilotić A., Nacka-Aleksić M., Pirković A., Bojić-Trbojević Z., Dekanski D., Krivokuća M.J. (2022). IL-6 and IL-8: An Overview of Their Roles in Healthy and Pathological Pregnancies. Int. J. Mol. Sci..

[58] Osmers R.G.W., Blaser J., Kuhn W., Tschesche H. (1995). Interleukin-8 synthesis and the onset of labor. Obstet. Gynecol..

[59] Shimoya K., Matsuzaki N., Taniguchi T., Okada T., Saji F., Murata Y. (1997). Interleukin-8 level in maternal serum as a marker for screening of histological chorioamnionitis at term. Int. J. Gynecol. Obstet..

[60] Ross K.M., Miller G., Culhane J., Grobman W., Simhan H.N., Wadhwa P.D., Williamson D., McDade T., Buss C., Entringer S. (2016). Patterns of peripheral cytokine expression during pregnancy in two cohorts and associations with inflammatory markers in cord blood. Am. J. Reprod. Immunol..

[61] Yockey L.J., Iwasaki A. (2018). Interferons and Proinflammatory Cytokines in Pregnancy and Fetal Development. Immunity.

[62] Creely S.J., McTernan P.G., Kusminski C.M., Fisher F.M., Da Silva N.F., Khanolkar M., Evans M., Harte A.L., Kumar S. (2007). Lipopolysaccharide activates an innate immune system response in human adipose tissue in obesity and type 2 diabetes. Am. J. Physiol.-Endocrinol. Metab..

[63] Sandler N.G., Koh C., Roque A., Eccleston J.L., Siegel R.B., Demino M., Kleiner D.E., Deeks S.G., Liang T.J., Heller T. (2011). Host response to translocated microbial products predicts outcomes of patients with HBV or HCV infection. Gastroenterology.

[64] Anderson K.V. (2000). Toll signaling pathways in the innate immune response. Curr. Opin. Immunol..

[65] Meheissen M.A., Patel V.B., Preedy V.R. (2022). Markers of Bacterial Translocation in Type 2 Diabetes Mellitus. Biomarkers in Disease: Methods, Discoveries and Applications.

[66] Moreno-Navarrete J.M., Ortega F., Serino M., Luche E., Waget A., Pardo G., Salvador J., Ricart W., Frühbeck G., Burcelin R. (2011). Circulating lipopolysaccharide-binding protein (LBP) as a marker of obesity-related insulin resistance. Int. J. Obes..

[67] Mokkala K., Pussinen P., Houttu N., Koivuniemi E., Vahlberg T., Laitinen K. (2018). The impact of probiotics and n-3 long-chain polyunsaturated fatty acids on intestinal permeability in pregnancy: A randomised clinical trial. Benef. Microbes.

[68] Pinto Y., Frishman S., Turjeman S., Eshel A., Nuriel-Ohayon M., Shtossel O., Ziv O., Walters W., Parsonnet J., Ley C. (2023). Gestational diabetes is driven by microbiota-induced inflammation months before diagnosis. Gut.

[69] Turjeman S., Collado M.C., Koren O. (2021). The gut microbiome in pregnancy and pregnancy complications. Curr. Opin. Endocr. Metab. Res..

[70] Yan M., Guo X., Ji G., Huang R., Huang D., Li Z., Zhang D., Chen S., Cao R., Yang X. (2023). Mechanismbased role of the intestinal microbiota in gestational diabetes mellitus: A systematic review and meta-analysis. Front. Immunol..

[71] Espinoza J., Romero R., Chaiworapongsa T., Kim J.C., Yoshimatsu J., Edwin S., Rathnasabapathy C., Tolosa J., Donnenfeld A., Craparo F. (2002). Lipopolysaccharide-binding protein in microbial invasion of the amniotic cavity and human parturition. J. Matern. Fetal Neonatal Med..

[72] Martinez-Lopez D.G., Funderburg N.T., Cerissi A., Rifaie R., Aviles-Medina L., Llorens-Bonilla B.J., Sleasman J., Luciano A.A. (2014). Lipopolysaccharide and soluble CD14 in cord blood plasma are associated with prematurity and chorioamnionitis. Pediatr. Res..

[73] López M., Figueras F., Coll O., Goncé A., Hernández S., Loncá M., Vila J., Gratacós E., Palacio M. (2016). Inflammatory Markers Related to Microbial Translocation Among HIV-Infected Pregnant Women: A Risk Factor of Preterm Delivery. J. Infect. Dis..

[74] Chen X., Scholl T.O. (2008). Association of elevated free fatty acids during late pregnancy with preterm delivery. Obstet. Gynecol..

[75] Zhang T., Jiang W.-R., Xia Y.-Y., Mansell T., Saffery R., Cannon R.D., De Seymour J., Zou Z., Xu G., Han T.-L. (2021). Complex patterns of circulating fatty acid levels in gestational diabetes mellitus subclasses across pregnancy. Clin. Nutr..

